# Role and mechanism of specialized pro-resolving mediators in obesity-associated insulin resistance

**DOI:** 10.1186/s12944-024-02207-9

**Published:** 2024-07-30

**Authors:** Xinru Liu, Yu Tang, Yuanyuan Luo, Yongxiang Gao, Lisha He

**Affiliations:** 1 College of Clinical Medicine, Chengdu University of Traditional Chinese Medicine, Chengdu, China; 2 College of International Education, Chengdu University of Traditional Chinese Medicine, Chengdu, China

**Keywords:** Obesity, Insulin resistance, Inflammation, Specialized pro-resolving mediator

## Abstract

With the changing times, obesity has become a characteristic epidemic in the context of the current era. Insulin resistance (IR) is most commonly caused by obesity, and IR is a common basis of the pathogenesis of many diseases such as cardiovascular disease, nonalcoholic fatty liver disease, and type 2 diabetes, which seriously threaten human life, as well as health. A major pathogenetic mechanism of obesity-associated IR has been found to be chronic low-grade inflammation in adipose tissue. Specialized pro-resolving mediators (SPMs) are novel lipid mediators that both function as “stop signals” for inflammatory reaction and promote inflammation to subside. In this article, we summarize the pathogenesis of obesity-associated IR and its treatments and outline the classification and biosynthesis of SPMs and their mechanisms and roles in the treatment of obesity-associated IR in order to explore the potential of SPMs for treating metabolic diseases linked with obesity-associated IR.

**Supplementary Information:**

The online version contains supplementary material available at 10.1186/s12944-024-02207-9.

## Introduction

Nowadays, obesity is considered an epidemic and has become a global health problem [1]. By 2030, 51% of Americans are predicted to be fat [2]. Metabolic abnormalities associated with obesity include a range of conditions such as dyslipidemia, diabetes, and cardiovascular disease, which have affected more than 650 million people worldwide [3]. Insulin resistance (IR) underlies the pathophysiology of these diseases. IR is a common pathological state characterized as a reduced reaction to stimuli by insulin in its primary target organs (e.g., skeletal muscle, adipose tissue, and the liver), which causes insulin to become unable to maintain appropriate lipid and glucose balance. As a result, insulin concentrations that are higher than normal levels are required to maintain normoglycemia [4–6]. Clinically, IR is often accompanied by obesity [7]. Chronic obesity causes systemic chronic low-grade inflammation and reduces insulin sensitivity, which is the main etiology of IR [1].

In addition to being driven by an array of proinflammatory mediators, inflammation is a crucial defensive mechanism of the host that is regulated by a set of self-limiting inflammatory mechanisms. As a result of these self-limiting mechanisms, inflammatory mediators, endothelial cells, and immune cells are recruited to eliminate proinflammatory mediators and inflammatory cells, restore harmed tissues, and promptly end the inflammatory response once inflammation has reached the proper stage. This process is referred to as inflammatory regression [8–12]. The regression of inflammation is an active process that accompanies the initiation of the inflammatory process [8]. However, the resolution process fails when inflammatory damage is severe and/or persistent, leading to excessive tissue injury and, eventually, chronic and self-promoting inflammation [11, 12].

Recently, when acute inflammation is in its regressive phase, anti-inflammatory and pro-lipolytic lipid mediators have been discovered that are produced endogenously from polyunsaturated fatty acids (PUFAs) derived from membrane phospholipids. These molecules, which are known as specialized pro-resolving mediators (SPMs), serve as both agents of the prompt resolution of inflammation and “stop signals” for the inflammatory response [13, 14]. SPMs can be derived from omega-3 PUFAs, which act by binding to G protein-coupled receptors [15]. SPMs control tissue homeostasis and inflammation via restricting neutrophils invasion into inflammatory foci, enhancing the cytotoxicity of apoptotic cells, decreasing inflammatory cytokines production, and promoting M2 macrophages polarization [16–19]. SPMs prevent the transition of inflammation to chronicity by stimulating neutrophil apoptosis and providing a mechanism mediated by endogenous agonists, which may remove the side effects caused by conventional anti-inflammatory agents [15]. SPMs provide an alternate method of treating inflammatory illnesses. SPMs have been tested in humans, as well as in animal models of inflammation [20]. Studies have shown that SPMs can alter neutrophil dysregulation and activation of phagocytic/lytic signaling in type 2 diabetes [21], reduce cardiometabolic risk factors such as inflammation and oxidative damage [22], and alter vascular function and thrombosis to prevent cardiovascular events [15].

Research indicates that SPMs generation in adipose tissue that is obese is inadequate and that supplementation with SPMs or omega-3 PUFAs, which produce these mediators, can reduce inflammation in obese adipose tissue [23–26]. Unlike conventional anti-inflammatory therapies, SPMs modulate macrophages, promote uptake and clearance of apoptotic cells, and improve inflammation resolution and tissue healing [25, 27]. SPMs act as endogenous lipid mediators that regulate inflammation and thus represent a unique strategy for treating inflammation. In recent times, there has been a surge in study on the roles and advantages of SPMs in diseases with chronic inflammatory (e.g., obesity, diabetes mellitus, and atherosclerosis) [28]. The aim of this paper is to summarize the potential mechanisms of action of SPMs in obesity-associated IR with a view to providing new therapeutic ideas and pathways for treating metabolic diseases associated with obesity-associated IR.

## Pathogenesis of obesity-associated IR

The etiology of IR includes genetic factors and environmental factors (e.g., aging, reduced physical activity, food intake, and smoking) [29–31]. As a risk factor, obesity significantly influences IR development [32]. Endoplasmic reticulum (ER) stress, oxidative stress, lipid deposition, inflammation, and mitochondrial dysfunction are all involved in the pathogenesis of obesity-induced IR [33].

Chronic obesity causes adipose tissue to become enlarged and dysfunctional, which recruits macrophages and polarizes them into a proinflammatory state [1]. One of the pathological features of obesity is the increased number of macrophages infiltrating adipose tissue [26]. Macrophages are considered to be a major source of proinflammatory mediators [34], and levels of proinflammatory adipokines including interleukin-6 (IL-6), monocyte chemotactic protein-1 (MCP-1), and tumor necrosis factor-α (TNF-α) are elevated in obesity and are connected with IR directly [35]. Conversely, the level of adiponectin with anti-inflammatory and insulin-sensitizing properties decreases [36, 37]. Overexpression of 5-lipoxygenase-activating protein is frequently observed in adipose tissue from patients and animals with obesity and IR [38, 39]. Leukotriene B4 (LTB4), a primary product of 5-lipoxygenase (5-LOX), activates nuclear factor-κB (NF-κB), thus increasing MCP-1 and IL-6 release [39].

Elevated plasma levels of free fatty acids (FFAs) are a major factor in IR in obesity [40]. In the setting of high FFA levels, stress sensors activate the inhibitor of NF-κB kinase (IKK) and c-Jun N-terminal kinase (JNK) pathways and their responses to downstream signaling cascade via classical receptor-mediated mechanisms [41]. By promoting insulin receptor substrate-1 (IRS-1) serine phosphorylation, activation of JNK and IKK in turn causes IR [41]. The downstream effector of these inflammatory pathways, namely, inducible nitric oxide synthase (iNOS), has been acknowledged as a crucial element in the feedforward mechanism that results in IR [33]. Obesity increases reactive oxygen species (ROS) production and induces lipid peroxidation in human adipocytes, liver, and skeletal muscle [42–44]. The equilibrium between antioxidant defense mechanisms and ROS is also affected by stress signaling pathways activation, such as the JNK [45] and NF-κB [46] pathways. ER stress is brought on by excessive unfolded/misfolded protein and lipids loads that build up in the ER during obesity and overnutrition [47, 48]. ER stress arises in the liver and adipose tissue via activation of JNK and inhibition of IRS-1 phosphorylation [48] and induces IR in endothelial cells. ROS and overloaded Ca^2+^ are the messengers of inflammation generated by ER stress, and the inflammatory state dictates when IR begins [1]. Excess fatty acids are deposited as ectopic fat in nonfatty organs including the pancreas, muscle, and liver. This produces lipotoxicity and dysregulation of organelles such as mitochondria and the ER, which releases excess ROS and proinflammatory factors. Moreover, insulin cannot function in insulin signaling pathways when there is persistent low-grade systemic inflammation, which also interferes with glucose homeostasis [1, 49]. In obese populations, mitochondrial fission in skeletal muscle increases, which leads to reductions in mitochondrial function and mass and thus to mitochondrial dysfunction and IR [50–52].

In addition, when the intestinal microbiota are dysbiotic, an increase in Gram-negative bacteria in the intestinal microenvironment produces large amounts of lipopolysaccharides (LPSs). High plasma levels of LPSs can induce a series of proinflammatory responses by activating toll-like receptor-2 (TLR2), TLR4, and TLR5 [53], which ultimately trigger obesity-associated IR. Exosomes derived from adipocytes are involved in macrophage activation by promoting M1 macrophages polarization and preventing M2 macrophages polarization, which in turn stimulates IR [54, 55]. The exosomes microRNA-27a [56], microRNA-29a [57], and microRNA-155 [58] can regulate insulin sensitivity through activating peroxisome proliferator-activated receptor-δ (PPARδ) or PPARγ.

## Treatments

Exercise-based lifestyle interventions can significantly reduce the chance of diabetes mellitus in obese insulin-resistant patients by promoting insulin secretion, increasing the sensitivity of tissues to insulin [59], and regulating lipid metabolism [60] to reverse the glycemic abnormalities associated with obesity-associated IR [61].

Biguanides inhibit gluconeogenesis by promoting the uptake of glucose by peripheral tissues [62]. Among these, metformin is most widely used in the clinical management of metabolic diseases. Metformin reduces obesity, decreases adipogenesis and gluconeogenesis, and increases glucose absorption in the liver, skeletal muscle, and adipose tissue [63]. Sodium-glucose cotransporter-2 (SGLT-2) inhibitors enhance the excretion of glucose in urine via blocking glucose reabsorption in the kidneys, which results in weight loss and antihyperglycemic effects [64]. Among these, by improving fat utilization and browning, empagliflozin inhibits weight gain. It also alleviates inflammation and IR induced by obesity by polarizing white adipose tissue (WAT) and M2 macrophages in the liver [65]. Glucagon-like peptide-1 (GLP-1) is an important enteric insulinotropic hormone that increases glucose-dependent insulin secretion, suppresses hepatic gluconeogenesis, and inhibits glucagon release [66]. Additionally, it results in decreased energy intake and appetite, as well as delayed stomach emptying [67, 68]. Various GLP-1 receptor agonists, including liraglutide and exenatide, may reduce body weight by reducing energy intake [69]. One of these, namely, semaglutide, also enhanced sensitivity to insulin, which may be due to overall weight loss [70]. Dipeptidyl peptidase-4 (DPP-4) is an essential incretin system regulator. It can be found in both soluble forms (sDPP-4) and membrane-bound forms. Studies have shown that adipokine sDPP-4 is related to metabolic inflammation [71]. DPP-4 inhibitors maintain blood glucose levels by preventing breakdown of the insulinotropic polypeptide that is glucose-dependent and GLP-1 [72]. By modulating the state of M1/M2 macrophage, DPP-4 inhibition by ligliptin reduces obesity-associated inflammation and IR [73]. Research in human and animal models reveals that the activation of NF-κB and IKKβ was considerably inhibited by nonsteroidal anti-inflammatory medications like salicylates and aspirin, which are cyclooxygenase (COX) inhibitors [74, 75]. Salicylates reduce levels of proinflammatory cytokines, decrease IR, and restore glucose homeostasis in type 2 diabetes by inhibiting IKKβ and NF-κB [76]. High-dose aspirin (6.77 ± 0.34 g) reduced fasting glucose levels, increased peripheral insulin sensitivity and glucose uptake in type 2 diabetes, probably by inhibiting IKKβ activity [77]. In addition, a salicylic acid derivative (600 and 900 mg triflusal) reduced levels of C-reactive protein in obese humans, increased insulin secretion induced by its action on β-cells, reduced fasting blood glucose levels, and improved glucose metabolism [78]. Salicylate (3 g/kg) suppressed systemic inflammation, decreased levels of insulin and fasting blood glucose, and alleviated IR by decreasing the amounts of leukocytes, neutrophils, and lymphocyte antigen 6 complex (Ly6C) in obese mice [79].

Although desired, changing one’s lifestyle has proven to be challenging [80]. There are also some side effects associated with medication. For example, SGLT-2 inhibitors can cause genital infections [81], and serious gastrointestinal events like nausea, vomiting, and diarrhea may become more common when GLP-1 receptor agonists are used [81, 82]. Although salicylates have been shown to be useful [77], complications associated with salicylates require attention, especially given the high dosages that are advised to treat diabetes. These include problems with the kidneys, excessive bleeding, gastric ulcers [33]. Despite the promise of anti-inflammatory approaches as adjunctive therapies, conventional anti-inflammatory treatments may cause adverse reactions and weaken the host’s defenses [25]. There is still a need to consider modulating inflammation while keeping the innate immune system active, which lowers the risk of infection. As a result, it’s imperative to continuously create novel therapeutic approaches.

## Synthesis and classification of SPMs

**Fig. 1:**
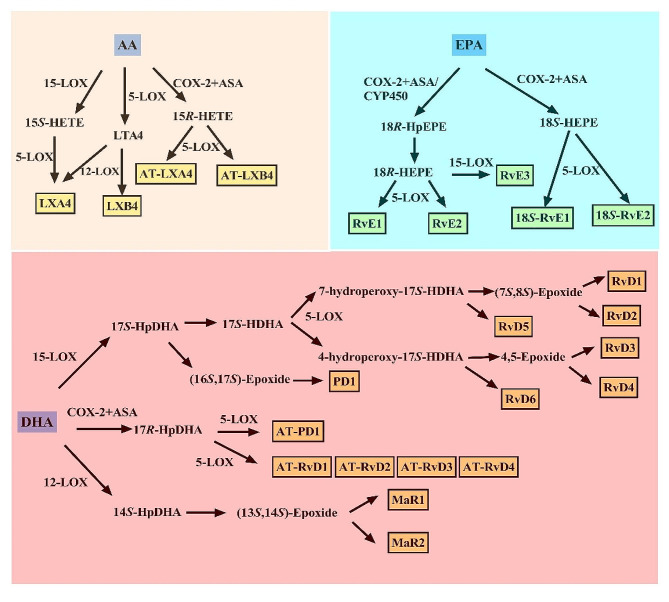
Biosynthesis of SPMs from omega-3 and omega-6 PUFAs. AA: arachidonic acid; ASA: aspirin acetylation; AT: aspirin-triggered; COX: cyclooxygenase; CYP450: cytochrome P450; DHA: docosahexaenoic acid; EPA: eicosapentaenoic acid; LOX: lipoxygenase; LTA4: leukotriene A4; LXA4: lipoxin A4; LXB4: lipoxin B4; MaR1: maresin 1; MaR2: maresin 2; PD1: protectin D1; RvD1: resolvin D1; RvD2: resolvin D2; RvD3: resolvin D3; RvD4: resolvin D4; RvD5: resolvin D5; RvD6: resolvin D6; RvE1: resolvin E1; RvE2: resolvin E2; RvE3: resolvin E3; 14*S*-HpDHA: (14*S*)-hydroperoxydocosahexaenoic acid; 15*R*-HETE: (15*R*)-hydroxyeicosatetraenoic acid; 15*S*-HETE: (15*S*)-hydroxyeicosatetraenoic acid; 17*R*-HpDHA: (17*R*)-hydroperoxydocosahexaenoic acid; 17*S*-HDHA: (17*S*)-hydroxydocosahexaenoic acid; 17*S*-HpDHA: (17*S*)-hydroperoxydocosahexaenoic acid; 18*R*-HpEPE: (18*R*)-hydroperoxyeicosapentaenoic acid; 18*R*-HEPE: (18*R*)-hydroxyeicosapentaenoic acid; 18*S*-HEPE: (18*S*)-hydroxyeicosapentaenoic acid

Omega-3 and omega-6 PUFAs are essential for SPMs biosynthesis, which include resolvins, protectins, and maresins produced from the omega-3 PUFAs eicosapentaenoic acid (EPA) and docosahexaenoic acid (DHA), as well as lipoxins (LXs) produced from the omega-6 PUFA arachidonic acid [13]. SPMs have an enzyme-dependent synthesis from PUFAs and exhibit picogram to nanogram activity [83]. The biosynthetic pathways of these novel lipid mediator molecules involve certain intermediates and the enzymes COX and LOX (Fig. 1) [13, 22, 28].

LXs were the first SPMs to be studied and described [84] and are formed by transcellular biosynthesis in the presence of multiple lipoxygenases [27, 83]. LXs include lipoxin A4 (LXA4), lipoxin B4 (LXB4), and their aspirin-triggered (AT) isoforms [22]. A classical pathway for the production of LXs is initiated by 15-LOX [85]. Arachidonic acid is transformed into (15*S*)-hydroxyeicosatetraenoic acid by 15-LOX and 5-LOX rapidly converts it into LXA4 [86]. Moreover, interactions between platelets and leukocytes can result in the biosynthesis of LXs. The enzyme 5-LOX in leukocytes forms the epoxide intermediate leukotriene A4, which is then transformed into LXA4 and LXB4 by platelet-derived 12-LOX [87].

Resolvins are divided into the D series produced by DHA and the E series activated by EPA [88]. The production of D-series resolvins is started by 15-LOX by converting DHA into (17*S*)-hydroperoxydocosahexaenoic acid (17*S*-HpDHA). This is rapidly reduced to (17*S*)-hydroxydocosahexaenoic acid (17*S*-HDHA), which 5-LOX then transforms into two hydroperoxide intermediates (7-hydroperoxy-17*S*-HDHA and 4-hydroperoxy-17*S*-HDHA) [13, 22, 25]. Of these, 7-hydroperoxy-17*S*-HDHA is either reduced by peroxidases to resolvin D5 (RvD5) or hydrolyzed to (7*S*,8*S*)-epoxides, which are subsequently converted into RvD1 or RvD2 [83]. In contrast, 4-hydroperoxy-17*S*-HDHA is involved in the synthesis of RvD6 or is reduced to the 4,5-epoxide to generate RvD3 and RvD4 upon enzymatic digestion [89]. The oxidation of EPA is the initial stage in producing the E series of resolvins. Aspirin acetylation of COX-2 or cytochrome P450 converts EPA into (18*R*)-hydroperoxyeicosapentaenoic acid (18*R*-HpEPE). Rapid reduction of oxygenated 18*R*-HpEPE to (18*R*)-hydroxyeicosapentaenoic acid (18*R*-HEPE) by 5-LOX can generate hydrogen peroxide, and 18*R*-HEPE is then converted into RvE1/RvE2 [90, 91]. Moreover, 18*R*-HEPE can also generate RvE3 via the action of 15-LOX [83, 90].

Protectin D1 (PD1) is a dihydroxyl-containing derivative of DHA that is generated by 15-LOX or aspirin acetylation of COX-2 [88, 92]. Firstly, 15-LOX transforms DHA into 17*S*-HpDHA [93]. This is then epoxidized to the (16*S*,17*S*)-epoxide, which is enzymatically hydrolyzed to produce PD1 [93].

Molecules of the maresin family are biosynthesized from DHA in macrophages [23]. In macrophages, DHA produces 14*S*-HpDHA via the action of 12-LOX, and 14*S*-HpDHA is hydrolyzed to produce maresin 1 (MaR1) and MaR2 after being enzymatically changed into the (13*S*,14*S*)-epoxide [94–96].

Aspirin-acetylated COX-2 that is generated as a result of the inhibition of prostaglandin synthesis by aspirin in cytokine-induced COX-2-containing cells transforms arachidonic acid into (15*R*)-hydroxyeicosatetraenoic acid. This is further converted by 5-LOX into 15-epimeric LXs (AT-LXA4 or AT-LXB4), which are also known as “aspirin-triggered” LXs [97]. DHA can also be changed into 17*R*-HpDHA through acetylation of COX-2 by aspirin, and 17*R*-HpDHA is then converted into the corresponding AT resolvins [83, 89] and AT PD1 by 5-LOX [23, 89]. Alternatively, EPA can be converted by aspirin acetylation of COX-2 into 18*S*-HEPE, which leads to producing 18*S*-RvE1 and 18*S*-RvE2 by 5-LOX [91].

## Mechanisms of SPMs in the treatment of obesity-associated IR

### SPMs can modulate adipokines/cytokines, reduce inflammation, and regulate glucolipid metabolism

Currently, it is commonly recognized that one of the main underlying mechanisms connecting obesity to systemic IR is persistent and uncontrolled inflammation [98]. One organ thought to be crucial in the formation of peripheral IR is WAT [99]. Lipids, enzymes, chemokines, cytokines, adipokines, microRNAs, and mRNAs are a variety of soluble bioactive molecules released by WAT, which have a critical effect on systemic immune responses, insulin sensitivity, and metabolism [100–102]. Proinflammatory adipokines (TNF-α, MCP-1, IL-6, and DPP-4) secreted by adipose tissue mediate the development of IR [103, 104]. Among these, by autocrine and paracrine mechanisms, the new adipokine DPP-4 can impair insulin sensitivity [105]. The expression of DPP-4 is increased in overweight and obese individuals’ visceral adipose tissue [106]. In addition, adiponectin and leptin are crucial for lipid and glucose metabolism. Both adiponectin and leptin can mediate different components of metabolic syndrome like diabetes, hypertension, and obesity, via various pathways such as regulation of systemic inflammation [107]. Central control of food intake and weight maintenance depend on leptin [108], and the literature also suggests that leptin exhibits proinflammatory properties via upregulating cytokines (e.g., IL-6) [109]. Adiponectin activation causes a decrease in IR and the control of many biological processes, such as immunity and inflammation [110].

Studies have shown that in obese mice (leptin receptor-deficient mice, mice with diet-induced obesity [DIO], and genetically obese mice) or in adipose explants, RvD1 (2 µg/kg for 8/16 days, 300 ng/mouse for 3 weeks, 10 nM) [98, 111–113], RvD2 (10 nM) [112], 17*S*-HDHA (a precursor of RvD1) (50 ng/g for 8 days) [114], MaR1 (2 µg/kg for 10/20 days, 50 µg/kg for 10 days, 1–10 nM) [115, 116], RvE1 (2 ng/g for 4 weeks, 1.2 ng/g for 4 days) [117, 118], PD1 (100/250 nM) [118], and LXA4 (1 nM) [119] either increased adiponectin expression or decreased inflammatory cytokines levels (e.g., MCP-1, TNF-α, and IL-6) and thus alleviated inflammation of adipose tissue. MaR1 (1–200 nM) reversed a TNF-α-induced increase in leptin levels in adipocytes derived from human subcutaneous preadipocytes [116]. RvD2 (10 nM) downregulated leptin secretion in WAT exosomes from obese mice [112]. SPMs can also regulate glucose and lipid metabolism. RvD1 (2 µg/kg for 8/16 days) reduced the levels of fasting blood glucose in leptin receptor-deficient (db/db) mice and had a tendency to lower levels of glycated hemoglobin [98]. RvD1 (300 ng/mouse for 3 weeks) reduced insulin and glucose levels, body weight, and cholesterol in DIO mice [111]. RvD3 (10 µg/kg for 8 weeks) decreased the weight in both body and liver of DIO mice, reduced hepatic accumulation of triglycerides, and lowered the homeostatic model assessment of insulin resistance (HOMA-IR) index and serum insulin levels [120]. MaR1 (2 µg/kg for 10 days) decreased the HOMA-IR index, insulin and fasting blood glucose levels, and the weight of subcutaneous fat in DIO mice [115]. MaR1 (0–10 µM) reduced palmitate-induced accumulation of triglycerides [121]. However, it is important to note that SPMs may act in a dose-dependent or a tissue-dependent manner. For example, the levels of adiponectin were elevated in epididymal white adipose tissue (eWAT) from DIO mice (2 µg/kg for 10 days) and leptin-deficient (ob/ob) mice (2 µg/kg for 20 days) [115], as were circulating adiponectin levels in DIO mice (35 µg/kg for 8 weeks) [121], after MaR1 treatment. This suggests that levels of this adipokine may be systemically affected by a high dose of MaR1 and/or a longer duration of MaR1 treatment. However, MaR1 (50 µg/kg for 10 days) had no significant impact on adiponectin levels in muscle and the liver [116]. Intraperitoneal injection of RvD1 (2 µg/kg for 8/16 days) decreased IL-6 expression in db/db mice’s adipose tissue [98], but RvD1 (10 nM) did not alter IL-6 levels in obese mice’s adipose explants [112]. In WAT explants from obese mice, RvD1 (10 nM) had no effect on leptin secretion, whereas RvD2 downregulated leptin [112]. The proinflammatory factor TNF-α upregulated DPP-4, whereas MaR1 (1–200 nM) blocked the upregulation of DPP-4 caused by TNF-α but increased DPP-4 expression in subcutaneous adipocytes of obese or overweight humans. This indicated that MaR1 would function better in an environment that promotes inflammation around adipocytes [116]. In summary, SPMs may be affected by the tissue, environment, dose, or other factors in vivo, which lead to differences in metabolic effects.

An important member of the fibroblast growth factor (FGF) family, namely, FGF-21, acts as an endocrine factor that is primarily expressed in the liver, although it is also present in the pancreas, muscle, and adipose tissue [122]. It is known that this peptide increases insulin sensitivity by decreasing gluconeogenesis in the liver and promoting absorption of glucose in adipocytes [123]. Protectin DX (PDX) (1 µM) generated a dose-dependent rise in FGF-21 expression. The inhibitory effects of PDX on phosphorylation of NF-κB and inhibitor of NF-κB caused by palmitate, as well as damage to insulin signaling, were eliminated by siRNA-mediated inhibition of FGF-21. PDX alleviated palmitate-induced hepatic inflammation and IR via FGF-21-mediated pathways [124]. However, MaR1 exhibited contrasting regulatory effects in another study. A study in DIO mice showed increased hepatic and circulating levels of FGF-21, and MaR1 (50 µg/kg for 10 days) reduced FGF-21 expression [125]. This decrease may have been secondary to a notable decline in liver fat content following treatment. It has been demonstrated that elevated FGF-21 levels are linked to fatty liver disease [126]. This increase is thought to occur in situations when there is increased carbohydrate and hepatic lipid signaling. In contrast, in mice lacking hepatic IRS-1 and IRS-2, strong hepatic IR reduced obesity and circulating levels of FGF-21 [127]. Furthermore, it is believed that obesity and IR contribute to the FGF-21-resistant state in rodents and humans [128, 129]. Therefore, the pathways by which SPMs regulate FGF-21-mediated obesity and IR may be an interesting object of study.

### SPMs can modulate macrophages and exert anti-inflammatory and pro-resolving effects

#### Reduction in macrophage recruitment and promotion of switching of macrophages to M2 phenotype

Adipose tissue in obesity has more infiltrating macrophages, and these form characteristic “crown-like structures” around necrotic adipocytes [130]. A vicious cycle comprising increased synthesis of proinflammatory mediators and massive recruitment of macrophages is sustained by infiltrating macrophages in inflamed adipose tissue [131–135]. The macrophages that are recruited function as classically activated macrophages (M1-like phenotype) expressing CD11b, CD11c and F4/80 as well as iNOS. These cells cause an abnormal release of proinflammatory adipokines (MCP-1, IL-6, IL-1β, TNF-α) that results in IR, along with adipocyte hypertrophy and/or hyperplasia [26]. As Lumeng et al. [136–138] suggested, the activation pattern of macrophages in adipose tissue recruited in obesity is comparable to that of M1-polarized macrophages. It was shown that both the number of CD11c-expressing M1-like adipose tissue macrophages and the ratio of M1 macrophages to M2 macrophages were associated with IR [133, 139]. Increased expression of M2 macrophage marker proteins such as chitinase-3-like protein (Ym1), arginase 1 (Arg1), IL-10, and CD206 indicates that macrophages polarization has shifted toward an M2-like phenotype, which is critical for adipose tissue homeostasis and inflammation regression [113].

SPMs can reduce macrophage counts in adipose tissue and alter their phenotype, which has been demonstrated in obesity-induced IR. In db/db mice, RvD1 (2 µg/kg for 8/16 days) in a nanogram dose reduced the formation of inflammatory macrophage-rich crowns in adipose tissue [98]. RvD1 (10 nM) and RvD2 (10 nM) reduced transadipose migration of monocytes and their adhesion to adipocytes driven by MCP-1 and LTB4 in human monocyte–adipocyte cocultures. This is a key process for recruiting macrophages and monocytes to inflamed adipose tissue [112]. Moreover, RvE1 (1.2 ng/g for 4 days) was demonstrated to decrease immunostaining of hepatic F4/80 in obese ob/ob mice [118]. Elevated LXA4 levels and reduced mRNA expression of IL-6 and F4/80 (two markers of inflammation) in eWAT were seen in transgenic mice that overexpressed arachidonic acid 5-lipoxygenase-activating protein (ALOX5AP) [140].

In addition to reducing macrophage recruitment, RvD1 (10 nM) upregulated a number of M2 macrophage indicators, such as Ym1, resistin-like molecule α, CD206, and IL-10, and greatly elevated Arg1 expression, a well-recognized M2 macrophage marker, while reducing the secretion of T helper cell cytokines generated by interferon γ/LPS. This resulted in a shift in macrophages polarization in adipose tissue from the M1 phenotype, which represents classically activated inflammatory features, to an anti-inflammatory state similar to M2 [113]. These outcomes agree with what Hellmann et al. reported [98], who found that RvD1 (2 µg/kg for 8/16 days) was able to alleviate IR in obese diabetic mice by raising the proportion of F4/80^+^ cells and decreasing the concentration of F4/80^+^CD11c^+^ macrophages in adipose tissues that expressed macrophage galactose-type lectin-1 (an M2 macrophage marker). An anti-inflammatory and pro-resolving effect was attributed to the recruited macrophages transition to the M2 phenotype [141]. Similarly, MaR1 (2 µg/kg for 10 days) decreased CD11c and F4/80^+^ cells expression in the WAT of DIO mice. MaR1 (2 µg/kg for 20 days) failed to alter macrophage recruitment but increased IL-10 and CD163 expression in ob/ob mice, two markers of M2 macrophages [115]. Activation of RvE1 receptor (ERV1) has shown potential to induce inflammatory regression by activating monocytes and macrophages to an M2-like phenotype. RvE1 binds to ERV1 (also known as ChemR23) on monocytes and macrophages and has a direct effect on immune cells [117].

#### Enhancement of phagocytic activity of macrophages

When leukocytes leave the site of inflammation or exude, they cross the peritoneal adipose tissue to reach the local lymph nodes [142]. Inflammation of WAT can be caused by excessive and persistent inflammation or by leukocytes that are activated in fat but are unable to reach the lymphatics during delivery of fat [13]. SPMs are considered to be potent modulators that inhibit the release of proinflammatory cytokines, block the activation and recruitment of neutrophils, and induce macrophages to activate their phagocytosis in a noninflammatory (anti-inflammatory) manner [22]. Macrophage phagocytosis of dead or apoptotic cells and cellular debris followed by exocytosis to the lymph nodes is a crucial additional step in reducing inflammation [8].

Within hours after inflammatory stimulation, SPMs act on leukocytes, endothelial cells, and epithelial cells through G protein-coupled receptors [143]. In polymorphonuclear neutrophils (PMNs), the expression of block lipid transport-1 and ERV1 is changed in type 2 diabetes patients [144]. Studies have shown that ERV1 activation [145, 146] reduces levels of inflammatory cytokines, enhances macrophage phagocytosis of apoptotic neutrophils [91], and inhibits neutrophil migration [147]. For example, PMN infiltration is decreased and uptake of PMNs by resolution-phase macrophages is restored in db/db diabetic mice when human ERV1 is overexpressed in transgenic (ERV1tg) mice [148, 149]. The human receptor ERV1 (CD11b promoter ligand-expressed) is overexpressed in ERV1tg mice in mature medullary-like cells, especially in response to proinflammatory stimuli [144]. The response to RvE1 was markedly enhanced in phagocytosis and cell killing by overexpression of ERV1 [148]. As Herrera et al. observed, phagocytosis, killing, and clearance of neutrophils in db/db mice were damaged in vivo and in vitro. After administration of RvE1 (10 and 100 ng/mL for 2 h), phagocytosis was stronger in db/ERV1 and ERV1 mice than in db/db and wild-type mice. Thus, RvE1 more effectively modulated the phagocyte phenotype of mice with overexpressed ERV1 (ERV1, db/ERV1), which resulted in inhibition of the accumulation of neutrophils and more effective elimination of bacteria [148]. RvD1 (10 nM) in nanomolar concentrations induced noninflammatory phagocytosis by macrophages in vitro, which is a critical stage in the process of inflammation resolution. In adipose tissue interstitial vascular cells, RvD1 also increases macrophages phagocytic activity [113].

There are also many reports of SPMs associated with macrophage phagocytosis, but these are not yet available in the case of obesity-associated IR, which still needs to be further explored. For example, MaR1 (100 nM) prompted phagocytosis and excretion by macrophages, which involved phagocytosis by macrophages to clear dead or apoptotic cells [150]. Apoptosis of PMNs is regulated by SPMs such as PD1 and LXA4 via upregulation of the expression of C-C chemokine receptor on T cells and apoptotic PMNs and stimulation of clearance of chemokines during lysis [151].

### SPMs can modulate inflammatory signaling pathways

AMP-activated protein kinase (AMPK) is an energy-sensing enzyme, and activation of AMPK inhibits obesity-induced inflammation via multiple molecular pathways [124]. It has been discovered that activating this kinase prevents proinflammatory cytokines from being produced, and it is considered to be a key regulator of the inflammatory response [152]. AMPK is well known to be a possible target for treating metabolic conditions including IR, type 2 diabetes, and nonalcoholic fatty liver disease [153]. AMPK was activated in adipose tissues from MaR1-treated (2 µg/kg for 20 days) ob/ob mice [115], and activated AMPK levels were elevated in adipose tissues from RvD1-treated (2 µg/kg for 8/16 days) db/db mice [98]. AMPK may be involved in mechanisms underlying the insulin-sensitizing or anti-inflammatory effects of RvD1 and MaR1. It has also been described how PDX promotes AMPK activation. AMPK is activated by PDX in an IL-6-independent manner. Expression of the hepatic factors selenoprotein P (SeP) and fetuin A is involved in hepatocyte insulin signaling. Treatment with PDX (1 µM) enhanced AMPK phosphorylation and silent information regulator 1 (SIRT1) expression and decreased palmitate-induced SeP and fetuin A expression (via AMPK/SIRT1-mediated forkhead box O1 and NF-κB, respectively), as well as IR in hepatocytes, via a pathway dependent on AMPK/SIRT1 [124].

The etiology of IR involves the activation of inflammatory signaling molecules (e.g., JNK and iNOS) [154]. PDX (1 µg) prevented lipid-mediated induction of iNOS in muscle and the liver, as well as hepatic activation of JNK via phosphorylation at Thr183/Tyr185 (phospho-JNK) [155].

Signal transducers and activators of transcription (STATs) are classical transcription factors that transduce signals triggered by cytokine receptors of type I and type II [156]. In the classical Janus kinase (JAK)-mediated pathway, phosphorylated JAK triggers the recruitment and phosphorylation of a STAT when an extracellular cytokine binds to its receptor. The STAT is then translocated to the nucleus after this activation, where it binds DNA elements and controls related genes transcription [157]. During type I interferon-induced reactions, hyperactivation of the IL-10 signaling pathway may inadvertently activate STAT1 and the inflammatory genes it targets (i.e., C-X-C motif chemokine ligand 9 [*CXCL9*] and *CXCL10*) [158]. When inflammatory human macrophages and obese visceral adipose tissue were treated with RvD1, the IL-10 pathway was prevented from being overactivated by decreasing STAT phosphorylation. RvD1 (1, 10, 50 nM) did not affect the anti-inflammatory response of IL-10 but inhibited STAT1, its target inflammatory genes such as *CXCL9*, and the activation of STAT3 [159].

An important regulator of the inflammatory reaction is the classical NF-κB pathway, which, when it exhibits unusual activation, increases downstream inflammatory factors expression including IL-1β, IL-6, and TNF-α [160], which is correlated with IR [161, 162]. PDX has been shown to reduce IR in skeletal muscle (0–1 µM, 1 µg/mouse for 8 weeks) [163] and adipocytes (2 µM) [164] via the NF-κB signaling pathway. PDX decreased the nuclear translocation of NF-κB, phosphorylation of inhibitor kBα, and proinflammatory cytokines expression (e.g., TNF-α and MCP-1) [163, 164].

The mitogen-activated protein kinase (MAPK) signaling pathway is essential to inflammation [165]. Because of its participation in controlling the production of inflammatory mediators through transcription and translation, MAPK has been recognized as a possible anti-inflammatory therapeutic target. It has the ability to translate extracellular signals, including growth factors and stress, into intracellular signaling pathways activation [166]. RvD1 (1, 10, 50 nM) raised heme oxygenase-1 (HO-1) expression, which is a target gene of IL-10, via a mechanism reliant on p38 MAPK activation and thereby contributed to inflammation resolution. The same study determined that the p38 MAPK signaling pathway is the true mechanism by which RvD1 and IL-10 have additive anti-inflammatory effects in obese adipose tissue [159]. However, in transgenic animals, the role of SPMs was shown to be the reverse. It was shown that in ERV1 and db/ERV1 mice, RvE1 (10 ng/mL, 100 ng/mL for 2 h) reduced MAPK phosphorylation in transgenic animals [148].

The NOD-like receptor thermal protein domain-associated protein 3 (NLRP3) inflammasome is a crucial mediator that links inflammation of adipose tissue brought on by obesity to IR [167]. The assembly of the inflammasome leads to autoactivation of caspase-1, which hydrolyzes the proinflammatory cytokines IL-18 and IL-1β to release their mature forms [168]. The “danger signals” linked to endogenous and external metabolic stress, such as FFAs and their derivatives [169], adenosine triphosphate, and ROS, are sensed by NRLP3 inflammatory vesicles. A lack of inflammasome components is related to the prevention of obesity-associated IR in several animal models [170–172]. NLRP3 levels in visceral adipose tissue of epididymis were reduced in mice fed a high-fat diet (HFD) treated with RvE1 (2 ng/g for 4 weeks) [117].

### SPMs can modulate insulin signaling pathways and improve insulin sensitivity and glucose uptake

The regulation of several fates of cells, including differentiation, proliferation, and survival, is largely dependent on the phosphatidylinositol 3-kinase (PI3K)/protein kinase B (Akt) pathway [173]. The PI3K/Akt axis serves as a central link in the insulin pathway that regulates hepatic glycogen synthesis, gluconeogenesis, and lipid synthesis [174, 175]. PI3K mediates the action of insulin by transmitting signals from the insulin receptor to downstream targets [176]. When obesity occurs, IRS-1-induced PI3K-mediated signaling is impaired due to phosphorylation of serine residues in IRS-1 [177] and inhibition of IRS-1-mediated activation of Akt [178, 179]. Insulin-induced glucose uptake by muscle and fat is mediated by the translocation of glucose transporter type 4 (GLUT-4) from intracellular compartments to the plasma membrane [180, 181]. GLUT-4 translocation induced by insulin and subsequent uptake of glucose rely on the activation of PI3K/Akt [182, 183]. PPAR is a member of the nuclear ligand-activated transcription factor superfamily. PPAR regulates gene transcription that govern the uptake and storage of lipids, as well as the metabolism of lipoproteins, the differentiation of adipocytes, and the action of insulin [184]. Changes in PPARγ expression are often observed in IR [185], and activation of PPARγ leads to insulin sensitization [118]. It has been demonstrated that SPMs improve glucose homeostasis and insulin sensitivity in skeletal muscle, adipose tissue, and the liver via PI3K/Akt, GLUT-4, and PPARγ.

RvE3 (1.2 ng/g for 11 weeks) regulated the Akt phosphorylation in DIO mice’s adipose tissue while enhancing insulin-stimulated PI3K activity, GLUT-4 translocation, Akt phosphorylation, and absorption of glucose in 3T3L1 adipocytes and improving insulin sensitivity [186]. Similarly, MaR1 (2 µg/kg for 10/20 days, 50 µg/kg for 10 days, 0.1 nM) [115, 183], RvD1 (2 µg/kg for 8/16 days) [98], and LXA4 (1 nM) [119] were capable of increasing Akt phosphorylation stimulated by insulin in adipocytes or adipose tissue. Akt phosphorylation in db/db mice was slightly elevated by RvE1 (10 ng/mL for 2 h), whereas in db/ERV1 mice there was a notable decrease in Akt phosphorylation (Ser478). Akt phosphorylation (Ser473, Thr308) was significantly reduced in ERV1 and db/ERV1 mice upon treatment with 100 ng/mL RvE1 [148]. It can be seen that Akt phosphorylation is inhibited by RvE1 in mice that overexpress ERV1. On the other hand, RvE1 (1.2 ng/g for 4 days) achieved significant insulin sensitization by upregulating GLUT-4, IRS-1, PPARγ, and adiponectin in adipose tissues [118]. LXA4 (1 nM) upregulated GLUT-4 and IRS-1 and also exhibited the same effect [119].

Similarly, skeletal muscle is crucial in the development of IR [187]. MaR1 partially returns the skeletal muscle of DIO mice to its normal state of insulin-stimulated Akt phosphorylation [183]. But it must be noted that MaR1 (50 µg/kg for 3 h, intraperitoneal injection) did not increase the inhibitory effect of insulin on the activation of Akt in lean mice’s WAT and skeletal muscle. In contrast, MaR1 administered intragastrically for 10 days at a dose of 50 µg/kg enhanced systemic insulin sensitivity and reduced hyperglycemia induced by HFD in DIO mice [183]. PDX (0–1 µM, 1 µg/mouse for 8 weeks) alleviated impairment of IRS-1/Akt-mediated insulin signaling in the soleus muscle of HFD-fed mice and palmitate-treated differentiated C2C12 cells (myoblasts) [163]. In addition, White et al. showed that PDX (1 µg) prevented lipid-induced IR and enhanced skeletal muscle Akt phosphorylation at Ser473 [155]. RvD3 (10 µg/kg for 8 weeks, 0–200 nM) protected to some extent against the harmful effects of an HFD and palmitate on insulin signaling in skeletal muscle (increased Akt and IRS-1 phosphorylation expression) [120].

### SPMs can modulate ER stress

The unfolded protein response is triggered by ER stress and allows cells to accommodate environmental changes and sustain ER homeostasis [188]. However, a proapoptotic signaling cascade causes apoptosis when ER stress is elevated [189]. Various chronic diseases begin and progress as a result of prolonged ER stress [190]. In obese liver and adipose tissue, there is increased ER stress [48], which causes IR via various pathways [120]. AMPK reduces ER stress, in addition to maintaining energy homeostasis [191], and is essential for insulin signaling in skeletal muscle [192] and hepatic de novo lipogenesis [193]. AMPK directly regulates the activation of autophagy, which reduces ER stress [194]. It has been shown that RvD3 (10 µg/kg for 8 weeks, 0–200 nM) increases AMPK phosphorylation and alleviates ER stress via a mechanism that depends on autophagy, which leads to reductions in IR in skeletal muscle and hepatic steatosis [120].

It is known that one of the first and most critical steps in apoptosis mediated by ER stress is calcium imbalance in the ER [195]. Regulating calcium movement across the ER membrane requires sarco/endoplasmic reticulum Ca^2+^ ATPase (SERCA). SERCA activation in the ER during ER stress alleviates unbalanced calcium homeostasis and thereby reduces lipid accumulation and ER stress-induced hepatocyte apoptosis [196]. Activation of AMPK increases the activity of SERCA [197]. There are three different isoforms of SERCA (SERCA1–3), and in the liver, SERCA2b is the major isoform of SERCA2 [198, 199]. It was shown that MaR1 (35 µg/kg for 8 weeks, 0–10 µM) stimulated AMPK and reduced hepatic ER stress induced by lipids in both in vivo (DIO mice) and in vitro model via AMPK-mediated SERCA2b activation and thus improved lipid metabolism and alleviated hepatic steatosis [121].

In the setting of obesity and diabetes, hepatic gluconeogenesis is increased by ER stress in the liver [200]. Research has indicated that AMPK is crucial for maintaining glucose homeostasis in skeletal muscles and the liver [201, 202] by phosphorylating many transcription factors and enzymes that participate in metabolism. AMPK activation stimulates HO-1 expression in renal cells, endothelial cells, and macrophages [203, 204]. Heme is degraded by HO-1 into biliverdin, iron, and carbon monoxide, which are cytoprotective against various stresses, including oxidative stress [205]. HO-1 has been suggested as a therapeutic target for metabolic disorders mediated by ER stress [206]. Moreover, liver damage and diseases linked to ER stress can be prevented by overexpressing HO-1 [207, 208]. PDX (1 µg/DIO mouse for 8 weeks, 1 µM) inhibited hepatic gluconeogenesis by AMPK-dependently enhancing HO-1 expression, which in turn inhibited ER stress [188].

## Recent advances

### Dietary supplementation

Omega-3 PUFAs (DHA and EPA) are used to biosynthesize SPMs, and endogenous SPMs can be formed more easily when dietary supplements enriched in omega-3 PUFAs are taken [209, 210]. It has been shown that Omega-3 PUFAs alleviate obesity-associated inflammation and/or IR in animal models, in vitro, and in clinical trials.

Omega-3 PUFAs-restored fat-1 transgenic mice are rich in SPMs, and IL-6 and IL-1β levels, in addition to proinflammatory chemokines, were lower in HFD-fed fat-1 mice [211]. Furthermore, long-chain n-3 PUFA levels restoration prevented obesity-associated IR by reducing JNK and iNOS activation induced by lipids in the liver and muscle [211]. By enhancing insulin-stimulated expression of ³H-glucose transport, GLUT-4 translocation, and IRS-1, coculturing DHA-enriched (50 µM) macrophages with adipocytes was able to preserve insulin sensitivity [212]. Intraperitoneal injection of 17-HDHA (derived from DHA) (50 ng/g for 8 days) improved glucose tolerance (increased expression of adiponectin, PPARγ, and GLUT-4) and reduced inflammation by decreasing NF-κB activation in adipose tissues from DIO mice [114].

In a four-week randomized controlled study, the body mass index of obese female patients supplemented with omega-3 PUFAs (760 mg DHA + 920 mg EPA) did not change in comparison with the low-calorie diet group. The inflammatory state of subcutaneous, mesenteric, and omental fat was improved by supplementing with omega-3 PUFAs. Despite a greater improvement in the proinflammatory profile of the omentum, inflammatory factors, glucose, insulin, or the HOMA-IR index did not significantly change [213]. Treatment of obese patients with calanus oil (2 g/day) for 12 weeks decreased the HOMA-IR index and fasting insulin levels, but no difference in glycosylated hemoglobin levels was observed [214]. Abdominally obese adults supplemented with 2 g fish oil (120 mg EPA + 860 mg DHA) daily for 12 weeks significantly reduced the levels of glycogen synthase kinase-3β, which may be a potential mechanism for reducing IR. In addition, the higher was the systemic inflammation status, the more significant were the reductions in insulin levels and the HOMA-IR index [215]. Supplementation with fish oil (4.0 g/day) for eight weeks in type 2 diabetics who are obese and 1.6 g DHA + 3.2 g EPA daily for 3 months in obese women reduced the levels of TNF-α, triglyceride/high-density lipoprotein ratio, and HOMA-IR index. Nothing changed in body weight or body composition [216, 217]. Women and men were supplemented with 3 g and 4 g fish oil (DHA + EPA), respectively, in a two 7-week randomized, double-blind study. It was found that high-dose omega-3 PUFAs supplementation resulted in lower insulin resistance index values and insulin and blood glucose levels in women [218]. Interestingly, in another trial, obese nondiabetic men and women given 2 g fish oil (120 mg EPA + 860 mg DHA) for 12 weeks exhibited markedly decreased HOMA-IR index values and fasting insulin levels without gender variability [219]. It is evident that the role played by supplementation with omega-3 PUFAs is related to treatment duration and dose, patient group, and gender and still needs to be explored.

### SPM analogs

Many endogenous SPMs are unstable either biologically or chemically, which makes them inappropriate for use as medications. This problem can be solved by designing synthetic analogs or mimetics, i.e., exogenously administered sets of functional SPMs. Although there have been fewer studies of SPM analogs or mimetics for the treatment of obesity-associated IR, their use in related diseases has been reported and has thus demonstrated great therapeutic potential.

Benzo-LXA4 (a benzo-fused (15*R*)-stereoisomer analog), which is an analog of LXA4, has been shown to reduce obesity-induced liver and kidney damage. Benzo-LXA4 (10 pM) significantly increased macrophage expression of CD206. Benzo-LXA4 (1.7 ng/g) downregulated p62 and LC3-II levels (autophagy markers) in HFD-fed mice’s adipose tissue, lowered alanine aminotransferase level, and reduced hepatic deposition of triglycerides, glomerular dilatation, and tubulointerstitial deposition of collagen [220]. LXA4 analogs have protective effects in animal models of diabetes-related nephropathy and atherosclerosis. Benzo-LXA4 (1.7 µg/kg) alleviated inflammation and monocyte adhesion in the aorta of diabetic ApoE^−/−^ mice, significantly reduced plaque formation in the aortic arch, reduced creatinine clearance and levels of kidney injury markers (e.g., TNF-α and MCP-1), and alleviated glomerular dilatation and dilatation of the adherent stroma. Benzo-LXA4 (1 nmol/L) decreased the TNF-α-mediated activity of NF-κB [221, 222]. Benzo-diethynyl-(17*R*)-RvD1 methyl ester (BDA-RvD1, a synthetic analog of RvD1) decreased neutrophil infiltration in the lungs (1 µg/mouse), shortened resolution intervals in *Escherichia coli* peritonitis (100 ng/mouse), and stimulated phagocytosis in human macrophages (0.1 pM–10.0 nM) [223].

Clinical studies of SPM analogs are now in the early stages. In a randomized controlled trial, inhalation of the LXA4 analog (5*S*,6*R*)-LXA4 methyl ester (50 µg/2 mL normal saline) improved lung function in asthmatic children [224]. Administration of 0.1% (15*R*/*S*)-methyl-LXA4 (LXA4 analog) cream (5 g/each infant) to infants with eczema significantly reduced the severity of eczema, with no clinical adverse events [225]. Clinical trials of synthetic analogs of RvE1 for the treatment of dry eye are under way [226].

Modification of natural compounds to form more metabolically stable analogs or mimetics is a direction for future research, and it is expected that more therapeutic strategies targeting the associated pathways will be developed.

### SPM synthase genes

Because the synthesis of SPMs is regulated by enzymatic pathways (e.g., those involving LOX enzymes), defects in synthases may lead to failure of inflammatory regression. Therefore, research targeting synthases is worth undertaking, and gene therapy targeting SPM synthases is a promising strategy. The *ALOX5AP* gene encodes the protein 5-LOX [38]. Overexpression of *ALOX5AP* results in increased production of LXA4, decreased obesity, and prevention of HFD-induced inflammation and IR [140]. The arachidonate 5-lipoxygenase (*ALOX5*) gene encodes the 5-LOX enzyme, which catalyzes the conversion of EPA into 5-HEPE, enhances the induction of regulatory T cells by macrophages, and alleviates inflammation of adipose tissue [227]. The arachidonate 12-lipoxygenase (*ALOX12*) and arachidonate 15-lipoxygenase (*ALOX15*) genes have been shown to be connected to obesity phenotypes and possess the capacity to treat obesity (e.g., inflammation and IR) [228].

### Other relevant studies

One potential new source of lipid mediators for SPMs could be brown adipose tissue. Cold exposure increased levels of brown fat-derived PUFAs such as DHA, docosapentaenoic acid, and 12-HEPE [229]. Cold exposure was found to reduce IR and inflammation in DIO mice, primarily via stimulating MaR2 production from brown fat and targeting it to hepatic macrophages [230]. Similarly, cold stimulation promotes the production of the omega-3 PUFA 12-HEPE by 12-LOX in brown fat, which promotes glucose uptake in adipose tissue and skeletal muscle via activation of the insulin signaling pathway [231].

Bariatric surgery is an effective way to alleviate diabetes. One clinical study included morbidly obese (*n* = 29) and mildly obese nondiabetic (*n* = 15) subjects. Preoperatively, levels of SPMs derived from DHA (RvD3, RvD4, PD1) in morbidly obese subjects were significantly higher than that in slightly obese individuals, possibly as an attempt to counteract inflammation. SPMs levels did not significantly differ between morbidly obese nondiabetic patients (*n* = 16) and diabetic patients (*n* = 13). At 1 year after surgery, morbidly obese individuals exhibited significant weight loss. Nondiabetic patients’ 17-HDHA, PD1, and RvD3 levels were notably lower compared to pre-surgery levels, but levels of these SPMs were unchanged in post-surgery diabetic patients. After surgery, although the level of 14-HDHA was reduced in diabetic patients in remission, 14-HpDHA could be converted into MaR1, whereas it was not converted into MaR1 in patients not in remission. It is evident that whether diabetes is in remission after bariatric surgery is related to the ability to produce MaR1 [232].

## Potential drawbacks of SPMs in studies of obesity-associated IR

According to previous studies, SPMs are crucial to the treatment of obesity-associated IR. However, there are still some shortcomings. (1) Exosomes have been a research hotspot in recent years among the medical community, and their significant role in obesity-associated IR has been summarized [233], but current research on the effects of SPMs on exosomes in this disease has not been addressed. (2) There are a wide variety of SPMs, but many studies have emphasized the function of RvD1, RvE1, MaR1, LXA4, and PDX, and there have been few studies on other SPMs. (3) Some studies of SPMs have shown significant anti-inflammatory effects, but not in obesity-associated IR, which needs further validation. (4) SPMs act in a dose-dependent way that is tissue-specific, and their capacity to reduce inflammation and increase insulin sensitivity varies between SPMs. This therefore needs to be further explored.

## Summary and prospects

**Fig. 2:**
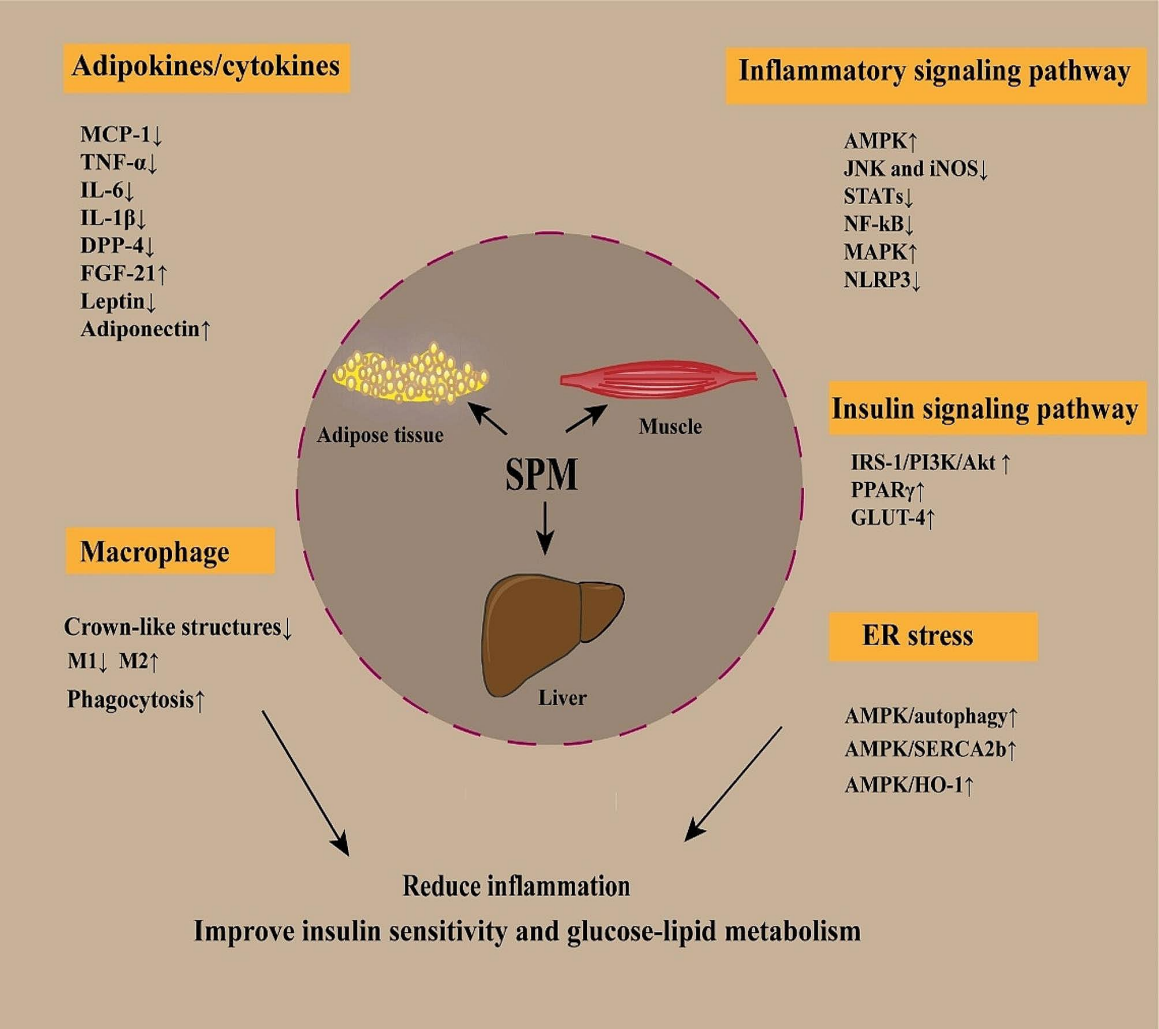
Mechanisms of SPMs in obesity-associated IR. SPMs can increase the expression of adiponectin and FGF-21, downregulate leptin and DPP-4, and decrease levels of inflammatory cytokines (e.g., MCP-1, TNF-α, and IL-6) and thus reduce inflammation. SPMs can also reduce the formation of macrophage crown-like structures, promote the switching of macrophages to the M2 phenotype, and enhance the phagocytotic activity of macrophages and thus exert anti-inflammatory and pro-resolving effects. In addition, SPMs regulate inflammatory signaling pathways, such as activation of AMPK and MAPK and inhibition of JNK, iNOS, STATs, NF-κB, and NLRP3. Moreover, SPMs can also regulate insulin signaling pathways and improve insulin sensitivity and glucose uptake by activating IRS-1/PI3K/Akt and promoting the expression of PPARγ and GLUT-4. In addition, SPMs activate AMPK and regulate ER stress via the AMPK/autophagy, AMPK/SERCA2b, and AMPK/HO-1 pathways and thereby alleviate IR.

Chronic inflammation is the primary mechanism of obesity-associated IR, and, because SPMs inhibit inflammation in a manner that does not compromise the defenses of the host, they may be promising and safe alternative treatments. There are a wide variety of SPMs, and there is still something to be added to the study of potential mechanisms of obesity-associated IR, in addition to those summarized above (Fig. 2). It is necessary to translate the results of research on rodent models of obesity to people, and further elucidation of the potential mechanisms of SPMs in obesity-associated IR may be important. Omega-3 PUFAs are substrates that produce SPMs, and omega-3 PUFA supplements have been clinically demonstrated to alleviate inflammation and/or IR associated with obesity. However, the doses of omega-3 PUFAs required for SPMs to achieve therapeutic effects and the duration of treatment are not yet standardized. On the other hand, the potential risks of using omega-3 PUFA supplements need to be noted, such as increased low-density lipoprotein levels [234]. Due to the instability of SPMs, SPM analogs have great potential. In the future, the dosage and treatment duration of dietary supplements, the development of SPM analogs, and more ways in which the utilization of SPMs can be increased deserve further research and exploration.

### Electronic supplementary material

Below is the link to the electronic supplementary material.

Supplementary Material 1

## Data Availability

No datasets were generated or analysed during the current study.
