# Supplementary material for: Role and mechanism of specialized pro-resolving mediators in obesity-associated insulin resistance
Source: Lipids Health Dis. 2024 Jul 30;23:234. doi: 10.1186/s12944-024-02207-9 (PMC11290132; doi:10.1186/s12944-024-02207-9)
Supplement: Supplementary file 1 — Supplementary Material 1 [file 12944_2024_2207_MOESM1_ESM.docx]

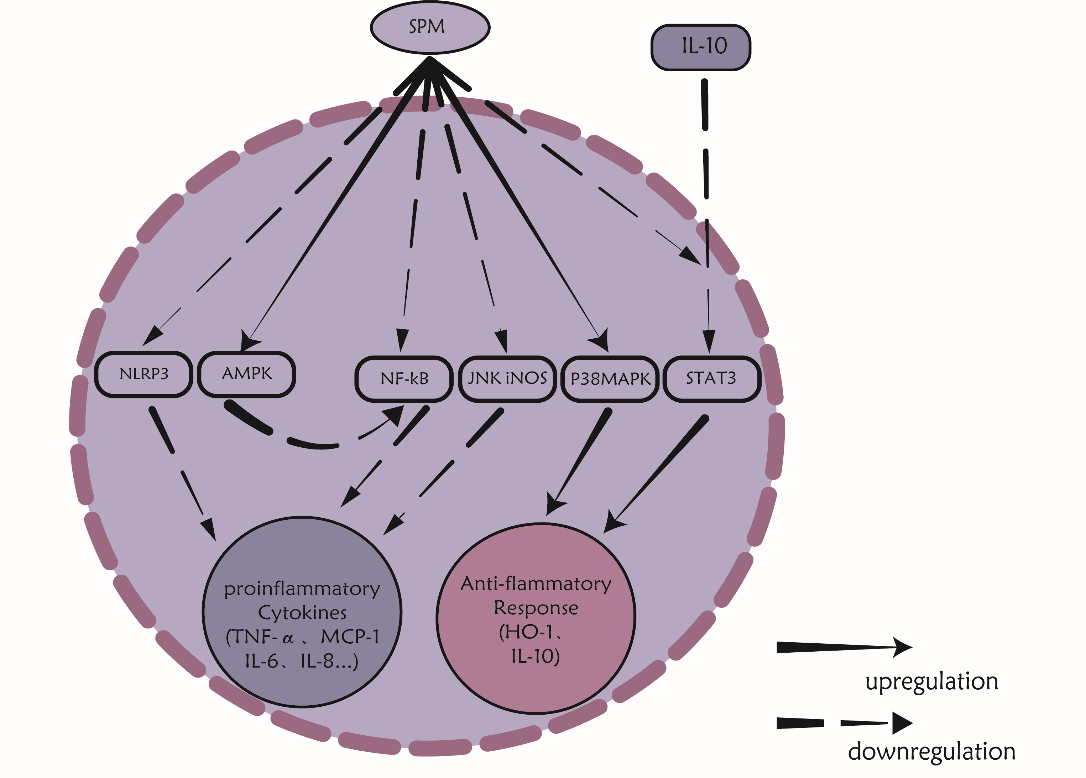


**Supplementary Figure 1.** SPMs regulate inflammation through signaling pathways


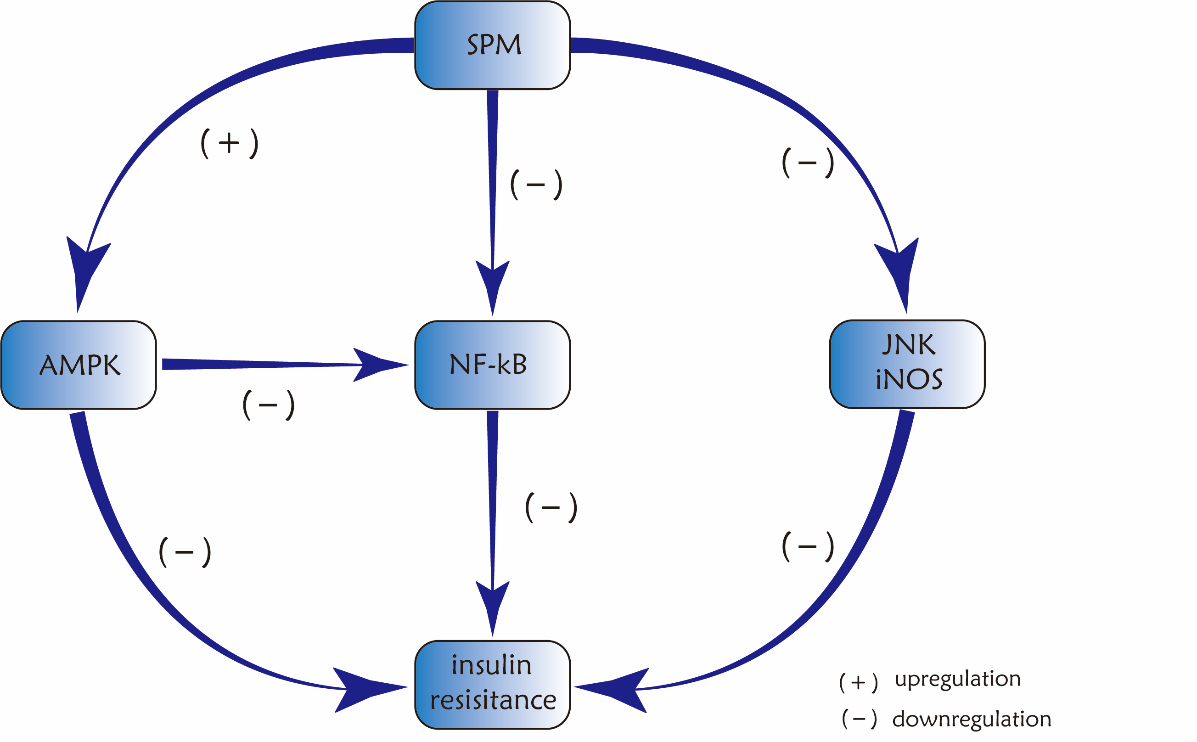


**Supplementary Figure 2.** SPMs regulate insulin resistance through signaling pathways

| **Supplementary Table**  Effects of SPMs on obesity-related IR in different models | | | | |
| --- | --- | --- | --- | --- |
| **SPMs** | **Model** | **Biological effects** | **Outcome** | **Reference** |
| n-3 PUFA | db/db mice  (fed containing 290 mg EPA/g oil and 190 mg DHA/g oil; 6 weeks) | Adiponectin↑ GLUT-4↑ PPARγ↑  fasting glucose↓  CLS ↓ F4/80↓  CD11c/CD206 ratio↓  MCP-1↓ | Attenuate adipose tissue inflammation and improve insulin sensitivity | [114] |
| 17-HDHA | C57BL/6J mice  (50ng/g; intraperitoneal injection; every 12 h for 8 days) | MCP-1↓TNF-α↓IL-6↓OPN↓  IκBα↑ NF-κB↓  CLS ↓CD11c/CD206 ratio↓  PPARγ↑ PPARα↑ GLUT-4↑ adiponectin↑  fasting insulin concentration↓ HOMA-IR↓ |  |  |
| DHA | C57BL/6J mice  (4μg/g; intraperitoneal injection; every 12 h for 8 days) | NF-κB ↓MCP-1↓TNF-α↓IL-6↓ OPN ↓  CLS↓CD11c/CD206 ratio↓  fasting insulin concentration↓  HOMA-IR↓ |  |  |
| ω-3 PUFA | ob/ob mice (5 weeks) | adipose tissue: adipokine↑ PPARγ↑  IRS-1↑ GLUT-4↑  hepatic: PPARγ↑IRS-2↑GLUT-2↑ | Alleviate obesity-induced insulin resistance and hepatic steatosis | [118] |
| DHA | ob/ob mice  (4 μg/g; intraperitoneal injection; every 12 h during 4d) | AMPK phosphorylation↑ |  |  |
| RvE1 | ob/ob mice  (1.2 ng/g; intraperitoneal injection; every 24 h during 4d) | adipokine↑ GLUT-4↑ IRS-1↑ PPARγ ↑ |  |  |
| PD1 | adipose tissue explants  (100 and 250nM; incubation for 12 h) | adipokine↑ |  |  |
| EPA and DHA | human adipose(100 μM DHA, 100 μM EPA, 50 μM DHA + 50 μM EPA; incubation for 48h） | IL-18↓IL-1β↓NLRP3↓ caspase 1 ↓ | Reduce inflammation | [121] |
|  | primary human adipocytes in coculture with THP-1 macrophages(100 μM DHA, 100 μM EPA, 50 μM DHA + 50 μM EPA; incubation for 48h) | IL-18↓ IL-1β↓ caspase 1 ↓  NLRP3（no change） |  |  |
|  | THP-1 macrophages in coculture with primary human adipocytes(100 μM DHA, 100 μM EPA, 50 μM DHA + 50 μM EPA; incubation for 48h） | IL-18↓IL-1β↓NLRP3↓caspase 1↓ |  |  |
| DHA | adipocytes co-cultured with DHA-enriched macrophages(50 μM for 5 days) | F4/80⁺/CD11⁺↓ IL-6↓TNFα↓  NF-κB↓  IL-10↑  GLUT-4↑ IRS-1↑ | Attenuate inflammation  and improve insulin sensitivity | [144] |
| DHA | obese mice  (4μg/g; ip; 10d) | MCP-1↓TNF-α↓IL-6↓IL-10↑  total number of macrophages（no change）  percentage of high CD11b/high F4/80 ↓  M2 markers:Arg1↑CD206↑Ym1↑ | Attenuate inflammation | [113] |
| RvD1 | elicited peritoneal macrophages  （0.1、1、10 and 100 Nm; incubation for 5h) | TNF-α↓ IL-6↓  M2 marker: Arg1 ↑  non-phlogistic macrophage phagocytosis↑ |  |  |
| LXA4 | adipose tissue explants (1 nM; incubation for 8h) | IL-6 ↓ IL-10 ↑  GLUT-4↑ IRS-1↑ | Attenuate inflammation  and improve insulin sensitivity | [119] |
| MaR1 | DIO mice  (2μg/kg; intraperitoneal injection; 10d) | F4/80-positive cells↓ CD11c ↓  Akt phosphorylation ↑  TNF-α↓ IL-1β↓ MCP-1↓  adiponectin↑ Glut-4↑ | Improve insulin sensitivity and attenuate adipose tissue inflammation | [115] |
|  | ob/ob mice  (2μg/kg; intraperitoneal injection; 20d) | CD163 and Il-10↑ (M2 macrophage markers)  MCP-1↓TNF-α↓ IL-1β↓DPP-4 ↓  adiponectin↑  insulin tolerance test ↑  Akt and AMPK phosphorylation↑ |  |  |
| MaR1 | hMSC-derived adipocytes  (0.1nM; incubated for  48 hours) | 2-Deoxy-D-glucose uptake and Akt phosphorylation ↑ | Reduce the hyperglycemia and the insulin resistance associated to obesity, at least in part by improving Akt signaling | [185] |
|  | lean mice  (50μg/kg; 3h; ip) | Akt phosphorylation in WAT and skeletal muscle（no changes） |  |  |
|  | DIO mice  （50μg/kg; 10d; oral gavage） | Akt phosphorylation in WAT in non-insulin-stimulated DIO mice↑  hyperglycemia↓  insulin tolerance test↑ |  |  |
| MaR1 | DIO mice  (50 μg/kg; 10d; oral gavage) | adiponectin↑ CT-1↑ FNDC5↑  DPP-4↓ | Reverse the expression of specific adipomyokines and hepatokines | [116] |
|  | human differentiated adipocytes  (1 and 10nM; incubated for 24h) | DPP4↑ CT-1↑ FNDC5↑  leptin↑adiponectin↑ |  |  |
|  | adipocytes treated with TNF-α  (1-200 nM; incubated for 24h) | DPP-4 ↓leptin↓  adiponectin↑ |  |  |
| MaR1 | DIO mice  (50μg/kg; 10d; oral gavage) | circulating FGF21 levels↓  hepatic: FGF21↓  WAT: FGF21↑ | Regulate FGF21 and contributes to its beneficial metabolic effects | [127] |
|  | primary hepatocytes  (0.1-10 nM; 6h) | FGF21↓  PPARα ↓ |  |  |
|  | HepG2 cells  (0.1-10 nM; 6h) | FGF21 ↓ |  |  |
| MaR1 | HFD-fed mice  (35μg/kg; intraperitoneally; 8 weeks) | AMPK phosphorylation↑  SERCA2b ↑  TG↓ ER stress↓ | Ameliorate hepatic steatosis via AMPK/SERCA2b-mediated suppression of ER stress | [123] |
|  | palmitate-induced primary hepatocytes  (0–10μm; 24h) | AMPK phosphorylation↑  SERCA2b ↑  TG↓ ER stress↓ |  |  |
| RvD1 | db/db mice  (2μg/kg; intraperitoneal injection; 8d/16d） | fasting blood glucose↓  HOMA-IR↓  Akt phosphorylation↑  CLS↓IL-6↓  the ratio of M2:M1↑  adiponectin↑  AMPK phosphorylation↑ | Resolve inflammation and enhance glucose tolerance and insulin sensitivity | [98] |
| RvD1 | adipose tissue explants (10nM; incubation for12h) | adiponectin↑ leptin↓TNFα↓ IL-12↓  IL-1β↓ MCP-1↓  adhesion of monocytes to adipocytes and monocyte transadipose migration↓ | Attenuate inflammation | [112] |
| RvD2 | adipose tissue explants (10nM; incubation for12h) | adiponectin↑leptin↓TNFα↓IL-6 ↓  IL-1β↓ MCP-1↓ IL-12↓  adhesion of monocytes to adipocytes and monocyte transadipose migration↓ |  |  |
| RVD1 | adipose tissue explants  (1,10,50nM; incubation for 30 minutes) | HO-1 ↑ IL-10↑  STAT3↓ STAT1↓  p38 MAPK↑ | Attenuate inflammation | [164] |
| RVD1 | C57BL/6J mice  (300ng/mouse; i.p; daily for 3 weeks) | leptin↓ adiponectin↑  JNK phosphorylation↓  serum insulin and glucose↓  number of macrophages↓  M1 markers↓ M2 markers↑ | Attenuate inflammation | [111] |
| RvD3 | obese mice  (10 μg/kg; [intraperitoneal injection](https://www.sciencedirect.com/topics/pharmacology-toxicology-and-pharmaceutical-science/intraperitoneal-injection); once every two days for 8 weeks) | IRS-1↑ Akt phosphorylation ↑  SREBP1↓ SCD1↓  ER sress↓  HOMA-IR↓ glucose tolerance↑  serum insulin levels↓ | Improve insulin resistance through AMPK/autophagy-associated attenuation of ER stress | [122] |
|  | C2C12 myocytes  (0–200 nM; 24h) | IRS-1↑ Akt phosphorylation↑ ER sress↓  AMPK phosphorylation and autophagy markers↑ |  |  |
|  | mouse primary hepatocytes  (0–200 nM; 24h) | SREBP1↓ SCD1↓ ER sress↓  AMPK phosphorylation and autophagy markers↑ |  |  |
| RvE1 | WT mice on HFD  (2ng/g; i.v.; twice weekly for 4 weeks) | TNF-α↓ IL-10↓ NLRP3↓  CD11b↓ | Control inflammation | [117] |
| RvE1 | db/ERV1 mice neutrophils (10 and 100 ng/ml; incubation for 2h) | neutrophil phagocytosis↑  Akt↓ (10 ng/ml)  Akt↓↓ (100 ng/ml)  macrophages↓  MAPK phosphorylation↓ | Rescue impaired neutrophil phagocytosis in obese T2D mice overexpressing ERV1 | [151] |
|  | db/db mice neutrophils (10 and 100 ng/ml; incubation for 2h) | Akt↑ (10 ng/ml ) |  |  |
|  | ERV1 mice neutrophils  (10 and 100 ng/ml; incubation for 2h) | neutrophil phagocytosis↑  Akt↓↓(100 ng/ml)  MAPK phosphorylation↓ |  |  |
| RvE3 | C57BL/6 mice  (1.2 ng/g; intraperitoneally; thrice per week for 11 weeks) | Akt phosphorylation↑  ITT↑ OGTT↑ HOMA-IR↓ | Improve insulin sensitivity and glucose tolerance | [188] |
|  | C2C12 myocytes  (0–200 nM; 24h) | Glut4↑PI3K↑  Akt phosphorylation↑ |  |  |
| PDX | db/db mice  （2μg; intravenously;  twice daily for 5 days） | IL-6↑  glucose infusion rate↑  IL-1β and TNF-α（no change） | Improve insulin sensitivity | [160] |
| PDX | human primary hepatocytes  (0–1μM; 24 h） | AMPK phosphorylation↑SIRT1↑Fetuin-A↓ SeP↓ | Reduce insulin resistance | [126] |
| PDX | differentiated C2C12 cells（0–1 Μm; 24 h） | Akt phosphorylation↑ IRS-1↑NF-kB↓IkBα phosphorylation↓AMPK phosphorylation↑PPARα ↑ | Reduce insulin resistance and inflammation | [157] |
|  | C57BL/6J mice  （1 μg/mouse/day; intraperitoneally; 8 weeks） | NF-kB↓IkBα phosphorylation↓TNF-α↓MCP-1↓AMPK phosphorylation↑PPARα ↑ glucose tolerance↑ |  |  |
| PDX | C57BL/6J mice  （1 μg/mouse/day; intraperitoneally; 8 weeks） | AMPK phosphorylation↑ HO-1↑  ER stress↓  blood glucose↓ | Suppress hepatic gluconeogenesis | [190] |
| PDX | adipocytes （0–2 μM; 24h） | AMPK phosphorylation↑NF-kB↓ IkBα phosphorylation↓ TNF-α↓ MCP-1↓ | Reduce insulin resistance and inflammation | [158] |
